# Green preparation and spectroscopic characterization of plasmonic silver nanoparticles using fruits as reducing agents

**DOI:** 10.3762/bjnano.6.27

**Published:** 2015-01-26

**Authors:** Jes Ærøe Hyllested, Marta Espina Palanco, Nicolai Hagen, Klaus Bo Mogensen, Katrin Kneipp

**Affiliations:** 1 Danmarks Tekniske Universitet DTU, Department of Physics and Department of Micro- and Nanotechnology, 2800 Kgs. Lyngby, Denmark

**Keywords:** fruits, green synthesis, luminescence, plants, plasmonics, SERS, silver cluster, silver nanoparticles

## Abstract

Chemicals typically available in plants have the capability to reduce silver and gold salts and to create silver and gold nanoparticles. We report the preparation of silver nanoparticles with sizes between 10 and 300 nm from silver nitrate using fruit extract collected from pineapples and oranges as reducing agents. The evolvement of a characteristic surface plasmon extinction spectrum in the range of 420 nm to 480 nm indicates the formation of silver nanoparticles after mixing silver nitrate solution and fruit extract. Shifts in plasmon peaks over time indicate the growth of nanoparticles. Electron microscopy shows that the shapes of the nanoparticles are different depending on the fruit used for preparation. The green preparation process can result in individual nanoparticles with a very poor tendency to form aggregates with narrow gaps even when aggregation is forced by the addition of NaCl. This explains only modest enhancement factors for near-infrared-excited surface enhanced Raman scattering. In addition to the surface plasmon band, UV–visible absorption spectra show features in the UV range which indicates also the presence of small silver clusters, such as Ag_4_^2+^. The increase of the plasmon absorption correlates with the decrease of absorption band in the UV. This confirms the evolution of silver nanoparticles from silver clusters. The presence of various silver clusters on the surface of the “green” plasmonic silver nanoparticles is also supported by a strong multicolor luminesce signal emitted by the plasmonic particles during 473 nm excitation.

## Introduction

Metal nanoparticles in various size ranges play an increasingly important role in many different fields of science, technology and medicine ranging from applications as catalyst, as antibacterial agents in medicine or plasmonic active structures in optical sensing and imaging [1–6]. These broad fields of applications generate a strong interest also in the preparation of metal nanoparticles. Many methods have been invented to synthesize nanoparticles, which can be mainly divided into top down and bottom up processes. Top down processes consist of physical processes where a solid is broken down into nanoparticles as it appears for example during laser ablation of nanoparticles from a macroscopic piece of metal [7–8]. Nanoparticles made by a physical process such as laser ablation have the advantage of being “chemically clean” with no impurities on their surfaces introduced by the chemical preparation process. In the bottom up approach, nanoparticles are created from even smaller structures such as silver ions, which are the outcome of a chemical process. The most popular process among the bottom up methods might be the preparation of silver and gold nanoparticles in aqueous solution by the reduction of silver and gold salts using sodium citrate or sodium borohydride as reducing agent [9]. Recently it has been identified that also plant extracts have the capability to reduce silver and gold salts and to create silver and gold nanoparticles [10–19]. Many different chemical compounds are present in various parts of different plants. Polysaccharides, phenolics, flavoids to mention only a few, could serve as reducing and also stabilizing agents [11]. Overall, using plant materials offers an eco-friendly way to prepare silver- and gold nanoparticles. Moreover, the diversity of chemical composition of plants, i.e., the combination of various reducing and stabilizing agents results in a broad variety of parameters in the chemical preparation process which enables synthesis of many different nanoparticles regarding size and morphology.

Here we study the formation of silver nanoparticles using fruit extracts from oranges and pineapples and check their capability as enhancing plasmonic structures for surface enhanced Raman scattering (SERS). Extracts from these two fruits have been used for preparing silver and gold nanoparticles [12,15–19]. Here we explore the formation of nanoparticles by varying conditions in the preparation process such as ratios of the mixtures of silver nitrate and fruit extracts and the presence or absence of light. Our studies focus on the preparation of nanoparticles between 10 and 100 nm, i.e., a size range which is of particular interest for plasmon-supported spectroscopy, such as SERS.

The formation of silver nanoparticles and their growth is monitored by the increase of the characteristic surface plasmon absorption around 420–470 nm and shifts of the absorption peak, respectively. Absorption signatures in the UV range which can be assigned to small silver clusters and surface enhanced luminescence spectra collected from the particles can provide information on the evolution process of silver nanoparticles.

## Results and Discussion

**Figure 1:**
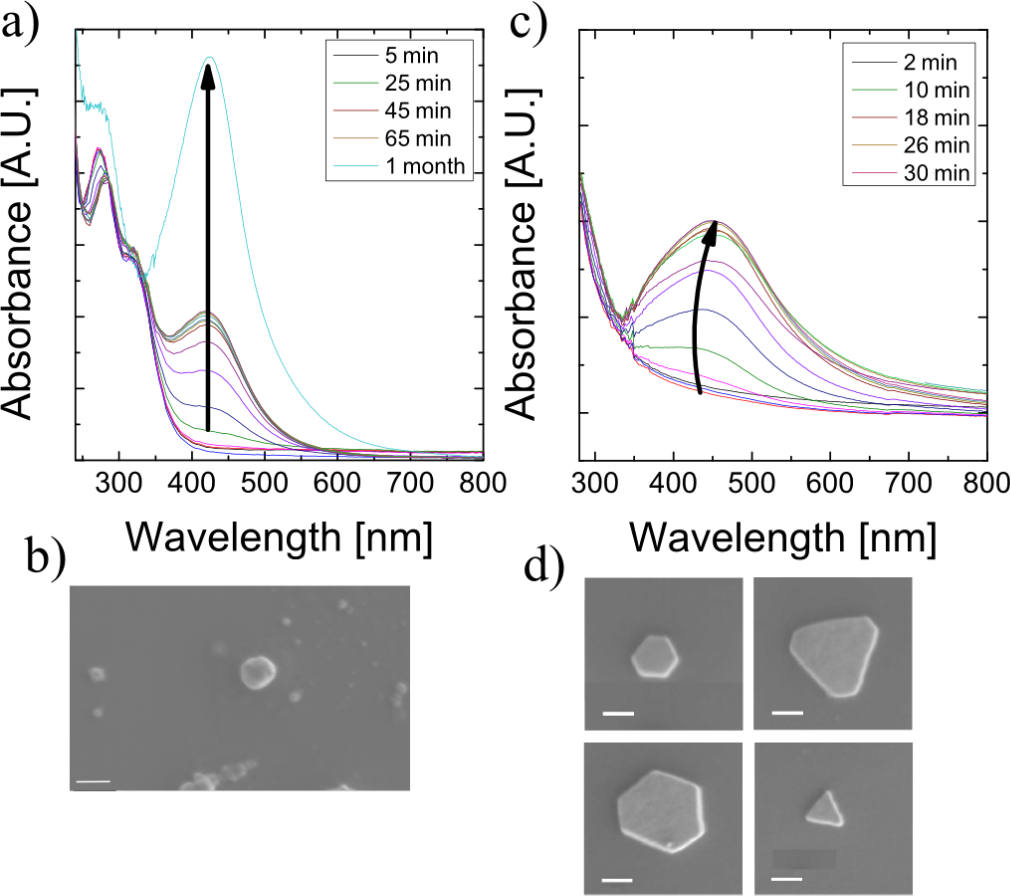
Time evolvement of UV–vis spectra of ≈1:10 orange extract/AgNO_3_ solution between 5 and 65 minutes after mixing and after 1 month (a) and of ≈1:50 pineapple extract/AgNO_3_ solution between 2 and 30 minutes after mixing (c). The arrows guide the eye between the peaks of the spectra. (b) and (d) show SEM images of typical nanoparticles prepared by using oranges and pineapples, scale bar is 100 nm.

Silver nanoparticles have been prepared at room temperature by mixing silver nitrate solutions and fruit extracts collected from pineapples and oranges. Evolution of the silver nanoparticles starts a few minutes after adding fruit extract to the AgNO_3_ solution and is indicated by the beginning evolvement of a characteristic surface plasmon extinction band. As Figure 1 shows, for orange extract, the peak of the plasmonic band appears at 420 nm with no shift during the growing process. This indicates that the particles are always within the Rayleigh limit (d << lambda). The increase of the absorption reflects the increasing number of such small silver particles over time. First indication of a plasmon band appears after about 30 minutes. The formation of particles can continue within time spans up to 1 month and longer. No extinction appears in the red and near infrared region.

For pineapple extract, the formation of silver nanoparticles is a faster process. The first sign of a plasmonic band can be seen already after 4 minutes and the process stops after 30 minutes. The peak of the plasmon spectrum appears at 450 nm and shows a small red shift over time. This shift indicates that for pineapple extract, not only the number of particles increases over time, but also the size and morphology of the particles change.

**Figure 2:**
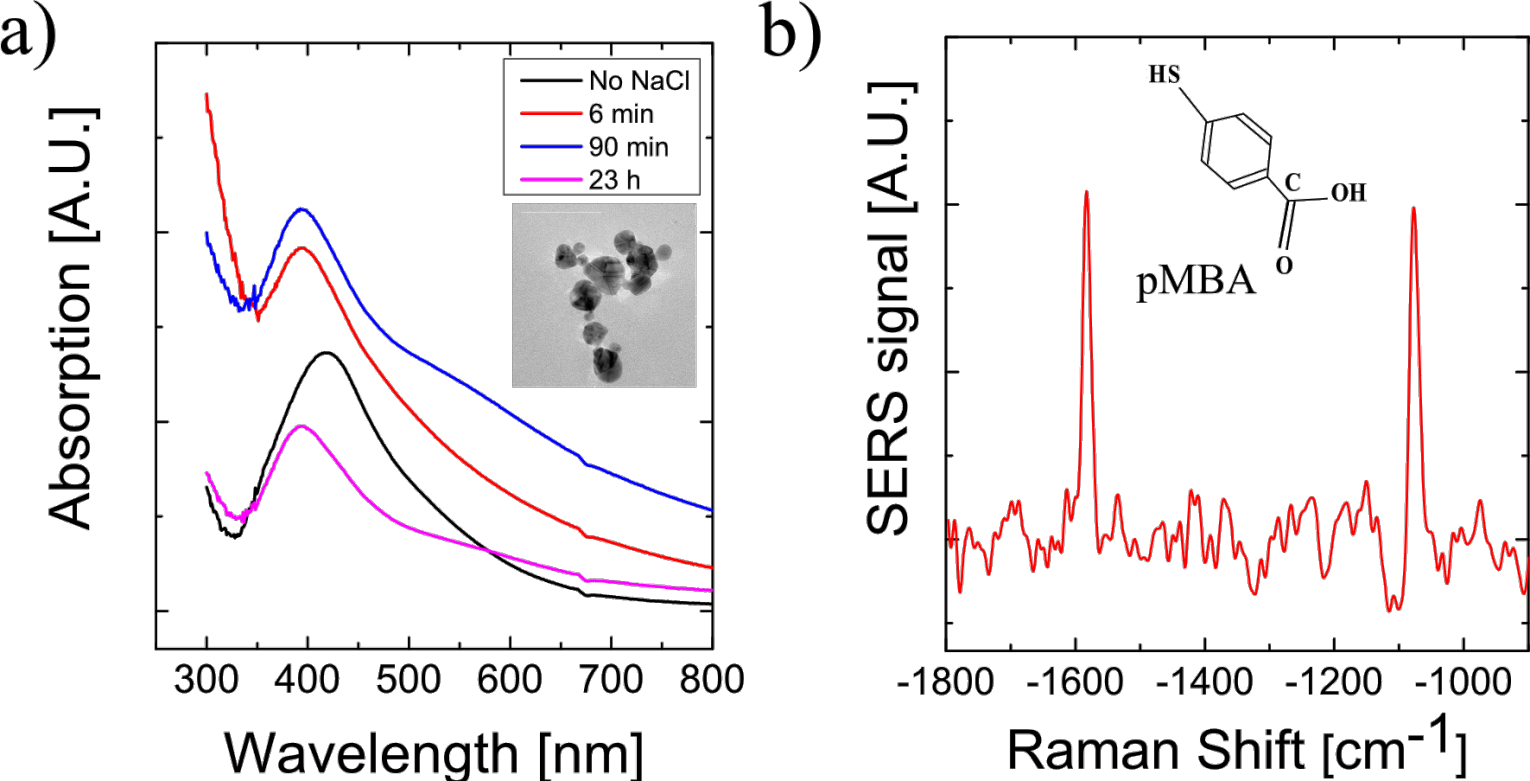
a) Extinction spectra of solutions of green silver nanoparticles made from orange extract before and after the addition of NaCl, final NaCl concentration was 0.2 M. The inset shows an aggregate formed by green nanoparticles. b) SERS spectrum of pMBA collected on green silver nanoparticles as enhancing nanostructure using 50 mW excitation at 785 nm.

An interesting property of green silver nanoparticles is their capability as enhancing plasmonic structures in SERS, particularly also when excitation in the NIR is used as in many biological applications [20]. High local fields can be generated in small gaps between nanoparticles such as in aggregates [21–23]. The green preparation process using orange extract delivers mainly isolated silver particles and aggregates with relatively wide gaps as it is shown for example in Figure 2a for nanoparticles made from orange extract. Experiments for creating aggregates of green silver nanoparticles with narrow interparticle gaps by adding various molecules, which are known for their capability to foster aggregation of silver nanoparticles [9], such as adenine, guanine, sodium chloride, failed. The extinction spectra in Figure 2a and the SEM image show that also NaCl at relatively high concentration does not force the formation of aggregates with subnanometer gaps as it had been obtained for other silver nanoparticles. The capability of green silver nanoparticles for enhancing Raman signals in SERS was tested by using NIR excitation and the non-resonant target molecule *para*-mercaptobenzoic acid (pMBA). Figure 2b shows a SERS spectrum of pMBA. The enhancement factor was inferred from a comparison with the normal Raman signal of the molecule. Using excitation in the NIR, the green silver nanoparticles provide only modest enhancement factors between 10 and 100 in agreement with computations of field enhancement factors for individual small silver spheres in the near infrared (NIR) [24]. The poor aggregation behavior of the green silver nanoparticles might be explained by the presence of other molecules on the surface of the particles related to plant materials introduced due to the green preparation, which prevent that particles come very close together and even touch each other. Moreover, these residual molecules might prevent that analyte molecules find optimum enhancing places in small distances to the surface of the plasmonic particles.

In general, the main differences in the outcome of the green preparation process are related to the different fruits as it is shown in Figure 1b,d. While orange extract gave rise to round and almost spherical silver particles, reduction by pineapple extract results in particles with sharp corners.

**Figure 3:**
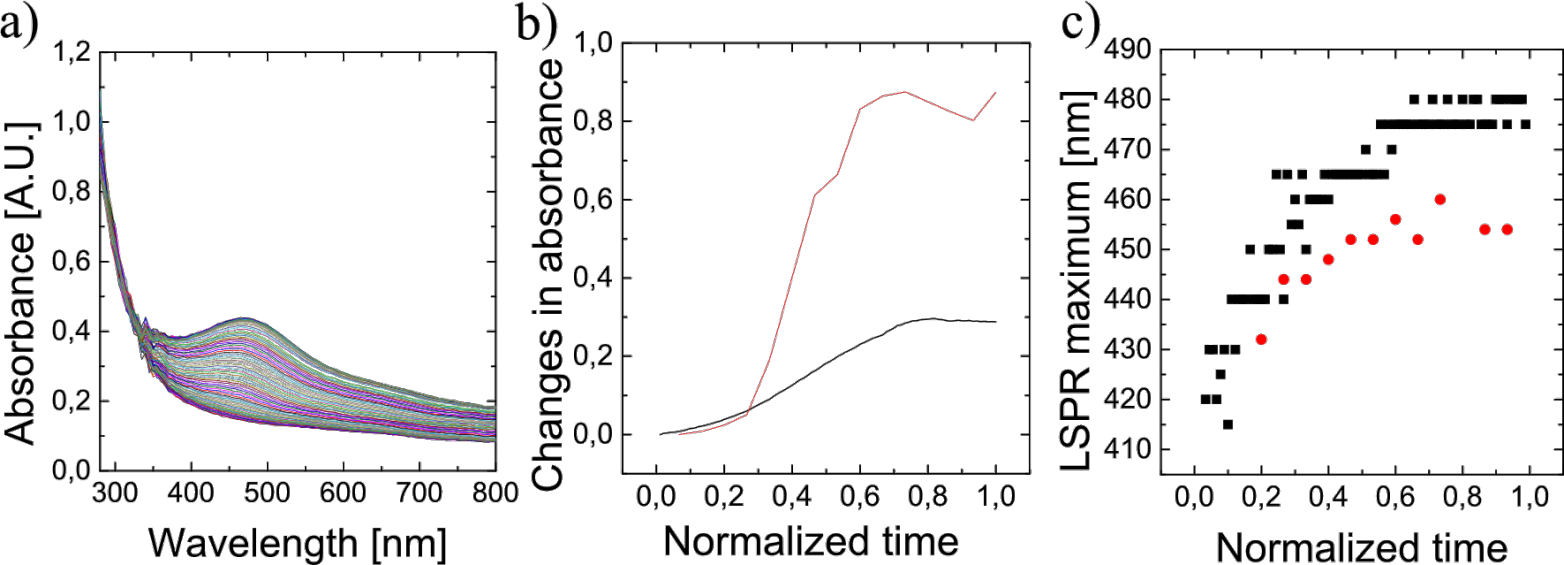
a) Time evolvement of UV–vis spectra of ≈1:50 pineapple extract/AgNO_3_ solution between 0 and 180 minutes after mixing in darkness, for comparison see Figure 1c that displays the same process in the presence of light. b) Peak growth taken from Figure 1c and Figure 3a as a function of normalized time. c) Peak position taken from Figure 1c and Figure 3a as a function of normalized time. Red and black data in Figure 3b,c are with and without light, respectively.

However, not only the kind of fruit determines size, shape and speed of growing of silver nanoparticles, also other experimental parameters during the green preparation process play a role. Figure 3 demonstrates the influence of light on the formation of silver nanoparticles using pineapple extract. Figure 3a shows the evolution of plasmonic particles under exactly the same conditions as used in Figure 1b. While the process shown in Figure 1b took place in the presence of light, silver particles in Figure 3a grew in the dark. Figure 3b shows the increase of the extinction over time in the presence of light and without light in a time scale normalized to the time for achieving the end of the reduction process, i.e., the time when no more increase of the extinction can be observed. The two curves show that the yield of the formation of nanoparticles is higher at the presence of light, but the time behavior in the normalized time scale is very similar. Figure 3c displays the peak position as a function of normalized time. Also this plot indicates that the chemical process with and without the presence of light might be almost the same. A difference of about 40 nm between the end peak positions in both processes indicates that the process without light resulted in larger nanoparticles.

**Figure 4:**
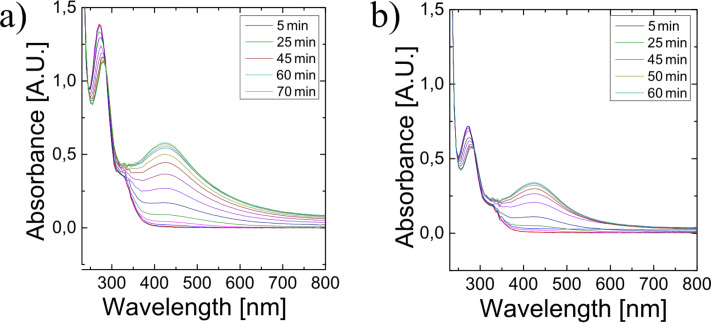
Time evolvement of UV–vis spectra in mixtures of orange extract and AgNO_3_ solutions between 5 and 60 minutes after mixing. Figure 4a,b represent different ratios of AgNO_3_ solution and orange extract with a 5 times higher ratio of the AgNO_3_ solution to plant extract in 4b than in 4a.

As an additional parameter in the preparation process, we change the volume ratio between the silver nitrate solution and the fruit extract. Figure 4 shows how different ratios of silver nitrate solution and orange extract influence the evolution of silver nanoparticles. The mixture used in Figure 4b was prepared with an excess of silver nitrate solution over the orange extract compared to Figure 4a. Figure 4 shows the surface plasmon peaks always at the same position at 420 nm, i.e., the size and shape of the nanoparticles do not depend on the ratio between silver nitrate and the reducing fruit extract. Much weaker plasmon bands in Figure 4b indicate that in this experiment, the fruit extract is the limiting factor for the total yield of nanoparticles.

**Figure 5:**
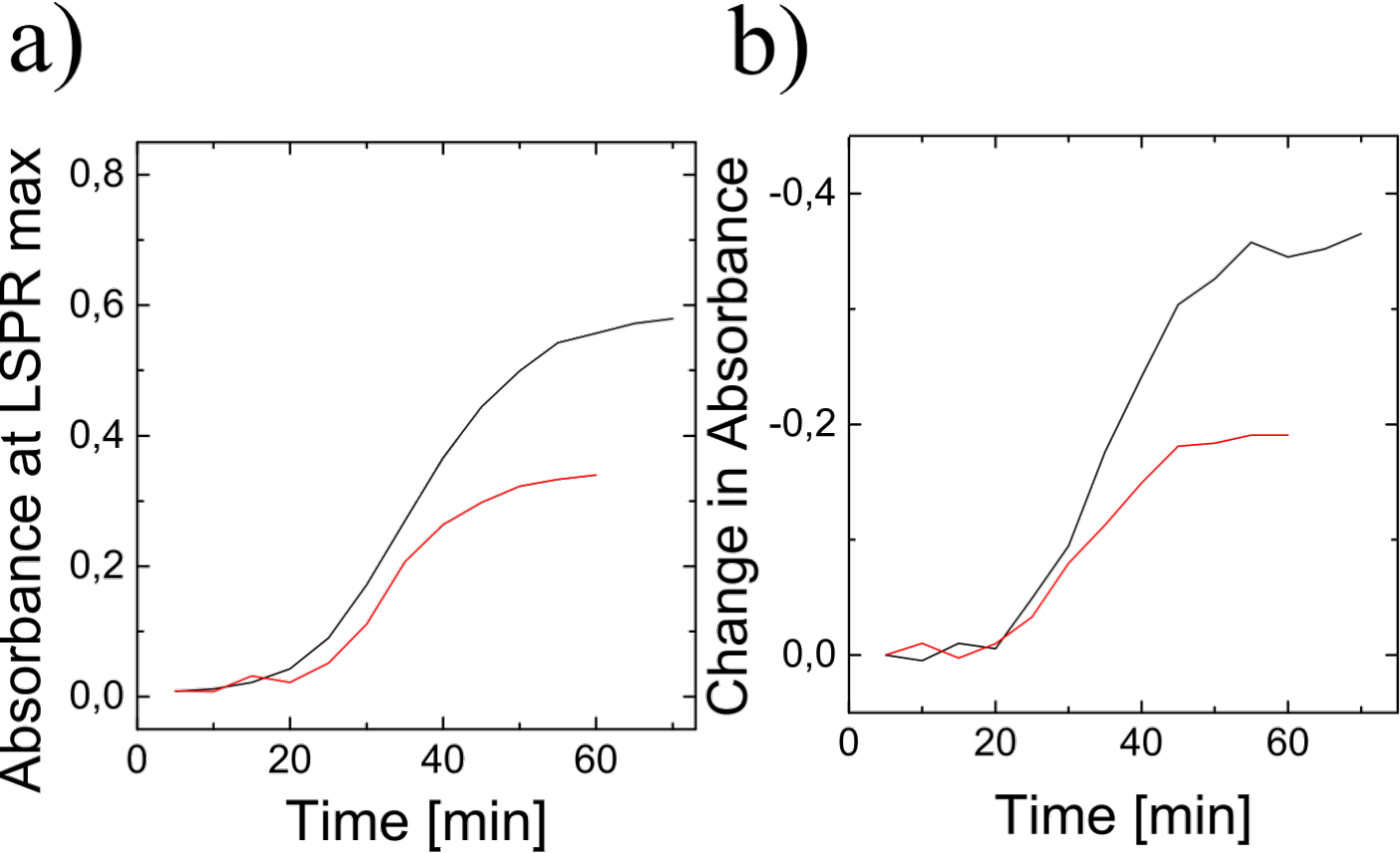
Time evolvement of the extinction spectrum during the formation of silver nanoparticles. The increase of the plasmon related peak at 420 nm (Figure 5a) correlates with the decrease of the peak at 270 nm related to Ag_4_^2+^ clusters (Figure 5b). Red and black curves represent different ratios of AgNO_3_ solution and orange extract.

In addition to plasmon resonances around 420 nm, the spectra displayed in Figure 4a,b and Figure 1a show peaks in the UV range, which are related to small silver clusters that can be formed from Ag^+^ available after the initial reduction process [25]. In particular, the observed UV band at 270 nm can be assigned to Ag_4_^2+^ clusters, which, via intermediates Ag_7_^3+^ and Ag_8_^+^, build up metallic particles Ag*_n_* [25]. Figure 5 shows that the increase of the plasmon absorption at 420 nm correlates with the decrease of absorption at 270 nm related to Ag_4_^2+^ confirming the conversion of silver clusters to plasmonic silver nanoparticles.

**Figure 6:**
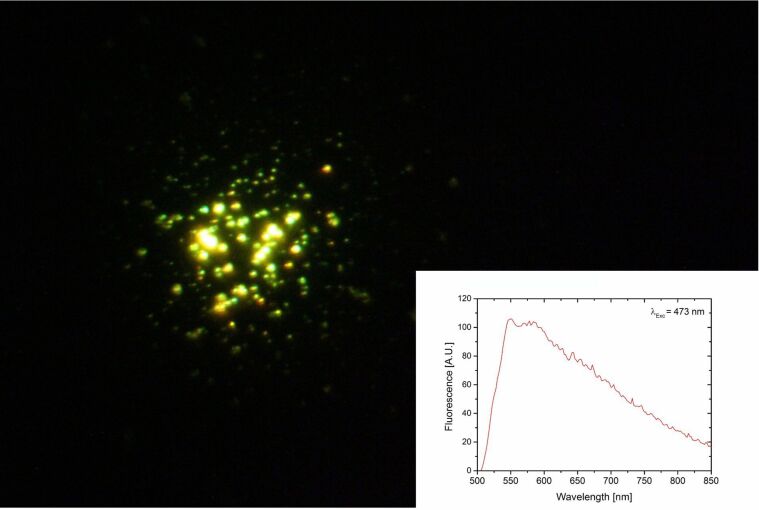
Image and spectrum of multicolor luminescence collected from green silver nanoparticles. The plasmonic silver particles were excited using 473 nm light provided by a 20 mW laser diode through a 100× oil immersion objective. The illuminated spot is around 10 µm^2^.

A strong luminescence signal in bright colors emitted from small silver clusters is a very interesting finding [26–27]. As it is shown in Figure 6, the presence of various silver clusters on the surface of the “green” plasmonic silver nanoparticles is also supported by a strong multicolor luminesce signal emitted by the plasmonic particles during visible excitation. The emission appears over a wide wavelength range with a strong contribution in the yellow-green region. At some places, the luminescent light shows strong fluctuations (see movie in Supporting Information File 1). These blinking hints to surface-enhanced emission of individual single emitters made from small silver clusters on the surface of plasmonic silver nanoparticles.

## Conclusion

Silver nanoparticles can be prepared in a relatively simple way by using extracts of oranges and pineapples as reducing agents. Size and shape of the particles depend mainly on the kind of fruit used in the chemical preparation process. The UV–vis absorption spectrum displays the surface plasmon resonance and also features in the UV, which can be assigned to small silver clusters. The correlation between the evolution of the surface plasmon band and the decrease of the silver cluster band in the UV confirms the conversion of silver clusters to plasmonic silver nanoparticles. Remaining single small silver clusters on the surface of the plasmonic nanoparticles give rise to a strong surface enhanced multicolor luminescence.

## Experimental

### Preparation of fruit extract

**Ananas comosus:** The pineapple extract was prepared by cutting away all parts except the yellow pulp of the pineapple. This was then sliced and blended after which the pulp was filtered through a standard coffee filter twice resulting in a yellow juice.

**Citrus sinensis:** The orange extract used in Figure 1 was prepared by taking an orange peel and washing it with demineralized water. Then it was cut into small pieces. 4 g of these pieces were added to 40 mL of demineralized water. This solution was then heated and stirred in a boiling water bath for 3 min. After this, it was cooled under stirring for 3 min and then filtered through a standard coffee filter followed by a 2 µm filter. The orange extract used in Figure 4 was made in exactly the same way with the difference that the 4 g of material were taken from only the white part of the orange peel.

All fruits were bought from the local supermarket. The pineapple came from South America. The oranges, sort “Valencia”, came from Italy, where they were grown without the use of pesticides.

## Supporting Information

A strong and fluctuating luminescence signal hints to surface-enhanced emission of individual single emitters on the surface of plasmonic silver nanoparticles.

File 1 Fluctuations in the luminescent light collected from green silver nanostructures (real time movie).
